# Donor genetic determinant of thymopoiesis rs2204985 impacts clinical outcome after single HLA mismatched hematopoietic stem cell transplantation

**DOI:** 10.1038/s41409-022-01751-1

**Published:** 2022-07-08

**Authors:** Chrysanthi Tsamadou, Sowmya Gowdavally, Uwe Platzbecker, Elisa Sala, Thomas Valerius, Eva Wagner-Drouet, Gerald Wulf, Nicolaus Kröger, Niels Murawski, Hermann Einsele, Kerstin Schaefer-Eckart, Sebastian Freitag, Jochen Casper, Martin Kaufmann, Mareike Dürholt, Bernd Hertenstein, Stefan Klein, Mark Ringhoffer, Sandra Frank, Christine Neuchel, Immanuel Rode, Hubert Schrezenmeier, Joannis Mytilineos, Daniel Fuerst

**Affiliations:** 1 Institute of Clinical Transfusion Medicine and Immunogenetics Ulm, German Red Cross Blood Transfusion Service, Baden Wuerttemberg—Hessen, Ulm, and University Hospital, Ulm, Germany; 2 Institute of Transfusion Medicine, University of Ulm, Ulm, Germany; 3 Department of Hematology/Oncology, University of Leipzig, Leipzig, Germany; 4 Department of Internal Medicine III, University of Ulm, Ulm, Germany; 5 Section for Stem Cell Transplantation and Immunotherapy, Department of Medicine II, Christian Albrechts University, Kiel, Germany; 6 Department of Medicine III, Johannes Gutenberg-University Mainz, Mainz, Germany; 7 Department of Hematology/Oncology, Georg-August-University Göttingen, Göttingen, Germany; 8 Department of Stem Cell Transplantation, University Hospital Hamburg Eppendorf, Hamburg, Germany; 9 DRST—German Registry for Stem Cell Transplantation, Ulm, Germany; 10 Department Internal Medicine I, Universitätsklinikum des Saarlandes, Homburg, Germany; 11 Department of Internal Medicine II, University Hospital Würzburg, Würzburg, Germany; 12 Medical Clinic 5: Hematology, Oncology, Nuremberg Hospital, Nuremberg, Germany; 13 Department of Medicine III, Hematology/Oncology/Palliative Care, Rostock University Medical Center, Rostock, Germany; 14 Department of Oncology and Hematology, Klinikum Oldenburg, University Clinic, Oldenburg, Germany; 15 2nd Department of Internal Medicine, Oncology and Hematology, Robert Bosch Hospital Stuttgart, Stuttgart, Germany; 16 Hematology/Oncology, Evangelic Clinic Essen-Werden, Essen-Werden, Germany; 17 Hematology/Oncology, Klinikum Bremen-Mitte, Bremen, Germany; 18 Universitätsmedizin Mannheim, Med. Klinik III, Mannheim, Germany; 19 Medizinische Klinik III, Städtisches Klinikum Karlsruhe, Karlsruhe, Germany; 20 ZKRD—Zentrales Knochenmarkspender-Register für Deutschland, German National Bone Marrow Donor Registry, Ulm, Germany

**Keywords:** Stem-cell research, Haematopoietic stem cells

## Abstract

A common genetic variant within the T cell receptor alpha (TCRA)-T cell receptor delta (TCRD) locus (rs2204985) has been recently found to associate with thymic function. Aim of this study was to investigate the potential impact of donor rs2204985 genotype on patient’s outcome after unrelated hematopoietic stem cell transplantation (uHSCT). 2016 adult patients were retrospectively analyzed. rs2204985 genotyping was performed by next generation sequencing, *p* < 0.05 was considered significant and donor rs2204985 GG/AG genotypes were set as reference vs. the AA genotype. Multivariate analysis of the combined cohort regarding the impact of donor’s rs2204985 genotype indicated different risk estimates in 10/10 and 9/10 HLA matched transplantations. A subanalysis on account of HLA incompatibility revealed that donor AA genotype in single HLA mismatched cases (*n* = 624) associated with significantly inferior overall- (HR: 1.48, *p* = 0.003) and disease-free survival (HR: 1.50, *p* = 0.001). This effect was driven by a combined higher risk of relapse incidence (HR: 1.40, *p* = 0.026) and non-relapse mortality (HR: 1.38, *p* = 0.042). This is the first study to explore the role of rs2204985 in a clinical uHSCT setting. Our data suggest that donor rs2204985 AA genotype in combination with single HLA mismatches may adversely impact post-HSCT outcome and should thus be avoided.

## Introduction

Six decades ago allogeneic hematopoietic stem cell transplantation (HSCT) revolutionized the treatment of otherwise incurable hematopoietic disorders and utmost malignant ones [1, 2]. The remarkable progress made in conditioning and graft vs. host disease (GvHD) prophylaxis regimens as well as in histocompatibility typing methods has undoubtedly improved the survival rates of transplanted patients [3]. Nonetheless, remaining morbidity and mortality rates are still important setbacks to overcome. Relapse of primary disease along with infection account for more than 60% of post-transplant mortality 100 days up to three years after allogeneic HSCT [4]. Incomplete T cell reconstitution as a result of impaired thymic recovery after HSCT has been shown to associate with poor clinical outcomes due to increased rates of infection, relapse and secondary malignancies [5, 6].

Thymus function is influenced by many factors but primarily by age and gender [7]. Particularly the age-related progressive atrophy of thymus known as thymic involution is a long described physiologic process [8, 9]. Although it does not lead to complete loss of function with some residual activity retained even in advanced ages, elderly patients do face higher risk of infection and relapse post-HSCT compared to younger ones due to a compromised thymic rebound after transplantation [10, 11]. Likewise, female gender has been linked with increased thymic output and slower progression of thymic involution [9]. This may partly account for the superior survival rates observed in female recipients compared to male ones irrespectively of donor gender in a large cohort of 12,000 patients [12].

Although strongly suspected, first significant evidence regarding the implication of genetic factors in thymic function and rate of involution came only a few years ago by Clave et al. [11]. After analyzing more than 5.5 million single-nucleotide polymorphisms (SNPs) they identified a common genetic variant (rs2204985) within the T cell receptor alpha (TCRA)-T cell receptor delta (TCRD) locus in the intergenic Dδ2-Dδ3 segments that was predictive of thymic function and T cell repertoire diversity. Particularly, in two independent cohorts it was shown that GG compared to the AA rs2204985 genotype correlated with a 43–44% increase of signal joint T cell receptor excision circles (sjTRECs), a surrogate marker of thymic output [11]. Furthermore, the same group reported that transplantation of rs2204985 AA human hematopoietic stem cells (HSC) into immunodeficient mice led to lower thymocyte counts as well as T cell receptor repertoire breadth [11]. Although the exact mechanism with which this genetic variation confers its effect on thymopoiesis remains unclear, the analysis results in the aforementioned humanized mouse model suggest that rs2204985 variant locally affects TCRD rearrangements. The findings of that study could find application in HSCT-donor selection as full T cell immune reconstitution after HSCT relies greatly on the de novo production of naïve T cells in the thymus of the recipient [6, 7]. During this process, lymphoid progenitors deriving directly from the graft or arising from the donor HSCs seed the host’s thymus where a bidirectional crosstalk between thymic stromal cells and developing thymocytes enables the formation of a broad but self-tolerant T cell repertoire [7]. Unfortunately, HSCT related factors like conditioning, opportunistic infections in the early post-HSCT period, glucocorticoids and GvHD adversely affect this procedure by directly damaging the sensitive thymic epithelium [7].

As of today, there are no published data regarding the potential impact of donor’s rs2204985 genotype on the outcome of unrelated HSCT (uHSCT). We hypothesize, based on the findings of the aforementioned humanized mouse HSCT model [11], that the graft’s rs2204985 genotype should have some impact on T cell reconstitution and subsequently on patient’s outcome after HSCT. Aim of this study is to investigate this hypothesis by retrospectively analyzing a large German cohort of unrelated HSC transplant pairs.

## Patients and methods

### Study population and clinical data

This study included a total of 2016 adult patients with hematologic malignancies (i.e. acute and chronic leukemia, MDS, NHL and myeloma) who received their first unrelated HSC graft (i.e. peripheral blood stem cells (PBSC) or bone marrow (BM)) between 2000 and 2013 in a German transplant center. Sample size was based on a-priori sample size calculation. Patients not achieving complete remission were not included in the cohort due to potential confounding by their increased disease burden and poor prognosis. Stem cell donor searches for cooperating transplant centers were conducted by the search unit in Ulm.

All clinical data were obtained from the German registry for stem cell transplantation (DRST), a subset of the EBMT ProMISe database for German patients. Patient consent was obtained for clinical data collection and registration in the EBMT database. Consent for histocompatibility testing in patients and donors was obtained upon initiation of the unrelated donor search. Treatment decisions along with follow up information from day 0, day 100 and yearly afterwards were collected by the cooperating transplant centers based on EBMT surveys (MED-AB-Survey). Missing data in the EBMT files was retrieved directly from the centers when possible. The study was approved by the ethical review board of the University of Ulm (project number 341/17).

### Definitions

The disease status prior to transplantation was classified according to definitions previously used by the EBMT study group [13]. Myeloablative conditioning (MAC) was defined according to the EBMT MED-AB manual Appendix III as well as published consensus suggestions [14]. Less intense regimens were considered as reduced intensity conditioning (RIC) [14].

### HLA and rs2204985 genotyping

High resolution HLA-typing (i.e. exons 2 and 3 for HLA-class I, and exon 2 for HLA-class II molecules) for the gene loci HLA-A, -B, -C, -DRB1, -DQB1 and –DPB1 was readily available. Only transplant pairs with maximum one single mismatch for the loci HLA-A, -B, -C, -DRB1 and -DQB1 (i.e. 10/10 or 9/10 HLA-matched) were included in the study. HLA-DPB1 mismatches were checked for permissiveness by applying the T-cell epitope (TCE) algorithm as previously described [15].

Genotyping of the rs2204985 in both patients and donors was performed by next generation sequencing (NGS) on an Illumina Miseq platform using DNA samples from unrelated donor search. The DNA sequence of the targeted intergenic region within the TCRA-TCRD locus for the design of the primers was retrieved from the NCBI SNP database [16].The oligonucleotide sequences of the forward and reverse rs2204985 specific NGS primers are as follows:

- fwd 5'-3': GCCTGAATTTAGCAACTGGGAGGAG

- rev 5'-3': GTTTCCCACTGAGGAGTTTGTCGGG

(Metabion International AG, Martinsried, Germany). Sequencing data analysis was carried out by the open source program for statistical computing “R”, version 4.1.2 [17].

### Outcome endpoints

Overall survival (OS), disease-free survival (DFS), non-relapse mortality (NRM), relapse, acute graft versus host disease (aGvHD) grade II-IV and chronic GvHD (cGvHD) were set as clinical outcome endpoints. Overall survival was defined as time to death from any cause or last follow-up. Disease-free survival was defined as time to treatment failure with death or relapse counting as events. Non-relapse mortality was defined as time from transplantation until any cause of death without previous relapse and disease relapse serving as competing risk. Relapse incidence was defined as time to the event of disease recurrence. This event was summarized by cumulative incidence estimate with death from other causes as the competing risk. The cumulative incidence of aGVHD grade II-IV, according to consensus grading [18], and cGVHD were calculated with death and disease relapse as competing risks. The clinical endpoints for the analysis in this study were defined according to the EBMT statistical recommendations [19].

### Statistical analysis

Statistical analysis of patient characteristics was performed by chi-squared test or fisher´s exact test for categorical and Mann-Whitney-*U*-test for continuous variables. For OS and DFS survival, Kaplan-Meier analysis with log-rank testing was used. Comparison of cumulative incidence for NRM, aGvHD, cGvHD and relapse was done using competing risks analysis as proposed by Fine and Gray [20]. Cox’s proportional hazards regression models was used for multivariate analyses of survival endpoints and competing risks regression was used for competing risks endpoints. All variables were tested for the affirmation of the proportional hazards assumption (PHA). Models were stratified for diagnosis and included adjustments for a center effect. A backward stepwise model approach was used to select variables for the respective endpoints with a threshold of 0.10 for retention in the model. No significant interactions between the tested variables (i.e. donor rs2204985) and the adjusted covariates were detected in any of the models. Significance level was set to *p* = 0.05. The open source program for statistical computing “R” (R Core Team), version 4.1.2 was used for all the statistical analyses.

## Results

### Cohort characteristics

The cohort consisted of 1392 10/10 (69.0%) and 624 9/10 (31.0%) HLA matched transplant pairs. With respect to rs2204985, three genotypes were identified (i.e. AA, AG and GG). Overall 25.7% (*n* = 519) of donors and 26.2% (*n* = 528) of patients carried the AA genotype. No differences regarding the donor rs2204985 genotype frequencies were observed between 10/10 and 9/10 HLA matched HSCTs. Furthermore, the distribution of other clinical predictors on account of donor rs2204985 genotype was similar within the 10/10 and the 9/10 HLA matched group, respectively. These data are summarized in Table 1. The donor rs2204985 genotype frequencies are presented in Table 2. Median follow-up time was 54.2 months.

**Table 1:** Cohort characteristics.

	10/10 rs2204985 AG/GG	10/10 rs2204985 AA	*P*-value	9/10 rs2204985 AG/GG	9/10 rs2204985 AA	Total	*P*-value
Patient age	53 (1–75)	54 (1–73)	0.527	51 (1–74)	52 (5–73)	2016	0.841
AML	426 (40.8)	157 (45.2)	0.479	199 (44)	74 (43)	856	0.529
ALL	153 (14.6)	46 (13.3)		74 (16.4)	29 (16.9)	302	
MDS	147 (14.1)	55 (15.9)		58 (12.8)	29 (16.9)	289	
NHL	114 (10.9)	31 (8.9)		30 (6.6)	15 (8.7)	190	
MM	54 (5.2)	20 (5.8)		32 (7.1)	6 (3.5)	112	
AL	64 (6.1)	15 (4.3)		26 (5.8)	5 (2.9)	110	
CLL	46 (4.4)	15 (4.3)		16 (3.5)	7 (4.1)	84	
CML	36 (3.4)	8 (2.3)		15 (3.3)	6 (3.5)	65	
HL	5 (0.5)	0 (0)		2 (0.4)	1 (0.6)	8	
Early stage disease	522 (50)	175 (50.4)	0.694	227 (50.2)	88 (51.2)	1012	0.359
Intermediate stage disease	314 (30)	97 (28)		138 (30.5)	44 (25.6)	593	
Advanced stage disease	209 (20)	75 (21.6)		87 (19.2)	40 (23.3)	411	
Donor age 18–30	364 (34.8)	114 (32.9)	0.507	141 (31.2)	48 (27.9)	667	0.857
Donor age 31–45	487 (46.6)	176 (50.7)		205 (45.4)	81 (47.1)	949	
Donor age 46–60	164 (15.7)	46 (13.3)		76 (16.8)	32 (18.6)	318	
Missing	30 (2.9)	11 (3.2)		30 (6.6)	11 (6.4)	82	
HLA-DPB1 permissive	614 (58.8)	213 (61.4)	0.390	240 (53.1)	94 (54.7)	1161	0.889
HLA-DPB1 non-permissive	426 (40.8)	134 (38.6)		210 (46.5)	78 (45.3)	848	
Missing	5 (0.5)	0 (0)		2 (0.4)	0 (0)	7	
Year of Tx 2000–2003	5 (0.5)	4 (1.2)	0.295	6 (1.3)	5 (2.9)	20	0.384
Year of Tx 2004–2009	524 (50.1)	165 (47.6)		262 (58)	100 (58.1)	1051	
Year of Tx 2010–2013	516 (49.4)	178 (51.3)		184 (40.7)	67 (39)	945	
In vivo T-cell depletion	691 (66.1)	249 (71.8)	0.069	291 (64.4)	109 (63.4)	1340	0.634
No in vivo T-cell depletion	224 (21.4)	69 (19.9)		81 (17.9)	36 (20.9)	410	
Missing	130 (12.4)	29 (8.4)		80 (17.7)	27 (15.7)	266	
Graft source PBSC	991 (94.8)	328 (94.5)	0.933	421 (93.1)	159 (92.4)	1899	0.897
Graft source bone marrow	54 (5.2)	19 (5.5)		31 (6.9)	13 (7.6)	117	
P-D CMV neg-neg	347 (33.2)	115 (33.1)	0.464	130 (28.8)	48 (27.9)	640	0.247
P-D CMV neg-pos	85 (8.1)	32 (9.2)		56 (12.4)	12 (7)	185	
P-D CMV pos-neg	237 (22.7)	84 (24.2)		123 (27.2)	58 (33.7)	502	
P-D CMV pos-pos	326 (31.2)	107 (30.8)		115 (25.4)	45 (26.2)	593	
Missing	50 (4.8)	9 (2.6)		28 (6.2)	9 (5.2)	96	
KPS 80–100	797 (76.3)	282 (81.3)	0.089	322 (71.2)	112 (65.1)	1513	0.207
KPS < 80	39 (3.7)	14 (4)		22 (4.9)	7 (4.1)	82	
Missing	209 (20)	51 (14.7)		108 (23.9)	53 (30.8)	421	
MAC	647 (61.9)	197 (56.8)	0.102	307 (67.9)	111 (64.5)	1262	0.479
RIC	398 (38.1)	150 (43.2)		145 (32.1)	61 (35.5)	754	

*AL* acute leukemia undifferentiated, biphenotypic or secondary, *M* match, *MM* mismatch, *PBSC* peripheral blood stem cells, *KPS* Karnofsky performance score, *MAC* myeloablative conditioning, *RIC* reduced intensity conditioning.

**Table 2:** rs2205985 genotype frequencies.

	HLA 10/10	HLA 9/10	Total	*P*-value
AA	347 (24.9)	172 (27.6)	519	0.095
AG	693 (49.8)	321 (51.4)	1014	
GG	352 (25.3)	131 (21)	483	

Hardy-Weinberg-Equilibrium test: HLA 10/10 *p* = 0.873, HLA 9/10 *p* = 0.422.

### Donor AA genotype adversely impacts survival after HLA single-mismatched HSCT

**Fig. 1:**
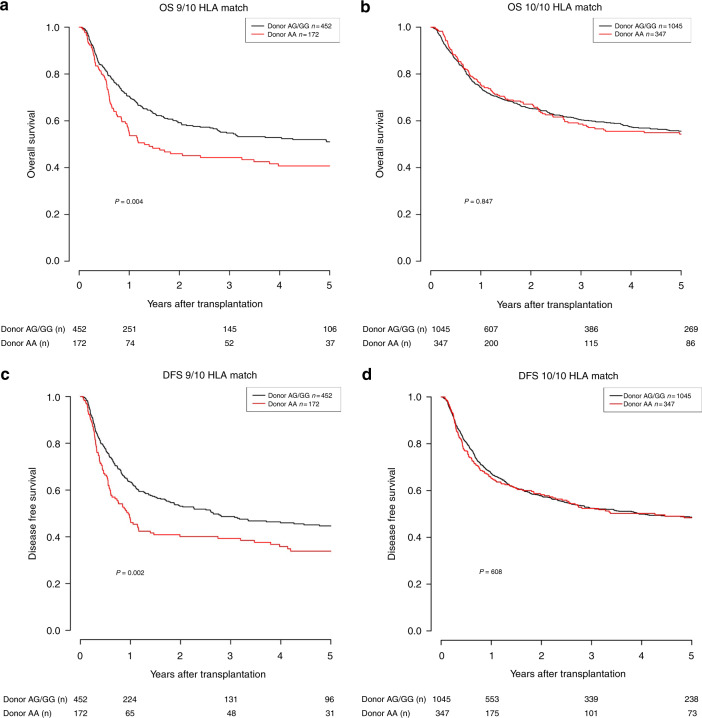
Univariate OS and DFS. **a** Overall survival (OS) according to donor rs2204985 in the subgroup of 9/10 HLA matched transplant pairs (*p* = 0.004). **b** Overall survival (OS) according to donor rs2204985 in the subgroup of 10/10 HLA matched transplant pairs (*p* = 0.847). **c** Disease-free survival (DFS) according to donor rs2204985 in the subgroup of 9/10 HLA matched transplant pairs (*p* = 0.002). **d** Disease-free survival (DFS) according to donor rs2204985 in the subgroup of 10/10 HLA matched transplant pairs (*p* = 0.608).

**Fig. 2:**
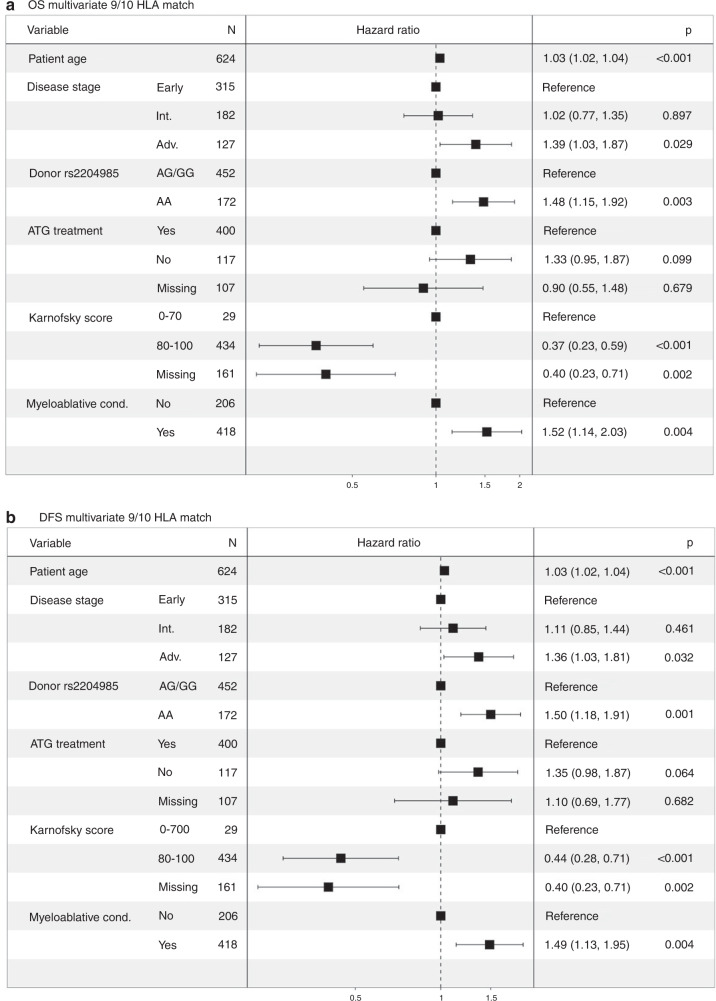
Multivariate OS and DFS. **a** Forest plot of multivariate analysis for overall survival (OS) in the subgroup of 9/10 HLA matched transplant pairs. **b** Forest plot of multivariate analysis for disease-free survival (DFS) in the subgroup of 9/10 HLA matched transplant pairs.

Regarding the effect of donor rs2204985 genotype on the primary outcome endpoints, weakly significant differences were observed in the DFS analysis (long-rank *p* = 0.024) between patients transplanted with AA and AG/GG grafts, while no statistical significance was reached as to OS (long-rank *p* = 0.121) for the complete cohort. Nevertheless, the respective Kaplan-Meier curves were indicative of a slightly worse outcome correlating with the donor AA genotype (see Supplementary Data 1, 2). Considering that HLA mismatch is a factor that profoundly influences the post-HSCT immunologic milieu of the recipient we analyzed the 10/10 and the 9/10 HLA matched cases separately. Indeed, analysis in the subgroup of single HLA mismatched cases (*n* = 624) revealed that donor AA genotype associated with markedly inferior OS (1Y after HSCT: 55.1% vs 70.6%; 5Y after HSCT: 40.7% vs 51%, long-rank *p* = 0.004, Fig. 1a) and DFS (1Y after HSCT: 47.6% vs 63.4%; 5Y after HSCT: 33.9% vs 44.6%, *p* = 0.002, Fig. 1c) after HSCT as compared to the donor AG/GG genotypes. These results were confirmed in the corresponding multivariate models (OS HR: 1.48, *p* = 0.003; DFS HR: 1.50, *p* = 0.001) which are visually displayed as forest plots in Fig. 2a, b, respectively. This detrimental effect of the donor rs2204985 AA genotype was not detectable in the fully HLA matched cases (see Fig. 1b, d). It is of note that the simultaneous survival analysis on account of donor rs2204985 genotype and HLA mismatch depicted in Supplementary 3 of the Supplementary Data suggests that the AG/GG donor genotype almost abrogates the adverse effect of HLA mismatch. Further results regarding the 10/10 HLA matched subgroup are presented in the Supplementary Data (Supplementary 4–7, Supplementary Table 1). Considering the known effect of age on thymic function we also conducted a further subanalysis regarding the effect of donor’s rs2204985 genotype on patient’s survival with respect to patient’s age. An age cut-off of 35 years was set. The latter was selected on the basis of optimal distribution of the two subgroups due to sample size considerations so that statistically sound analyses could be performed. The donor’s genotype appeared to have no effect on OS in patients aged less than 35 years and who received a single HLA mismatched graft. In contrast, the effect was markedly strong in the corresponding older subgroup (i.e. ≥ 35 y), *p* < 0.001. The respective Kaplan-Meier curves are presented in the Supplementary Data (Supplementary 8, 9). Another subanalysis with respect to patient’s gender revealed that the donor AA genotype markedly impacted the outcome of male patients (*n* = 369, HR: 1.76, *p* = 0.001) compared to that of female ones (*n* = 255, HR: 1.22, *p* = 0.364). The results of these multivariate models are presented in Supplementary Table 2A, B.

### AA genotype detrimental effect attributed to higher risk of relapse and NRM

**Fig. 3:**
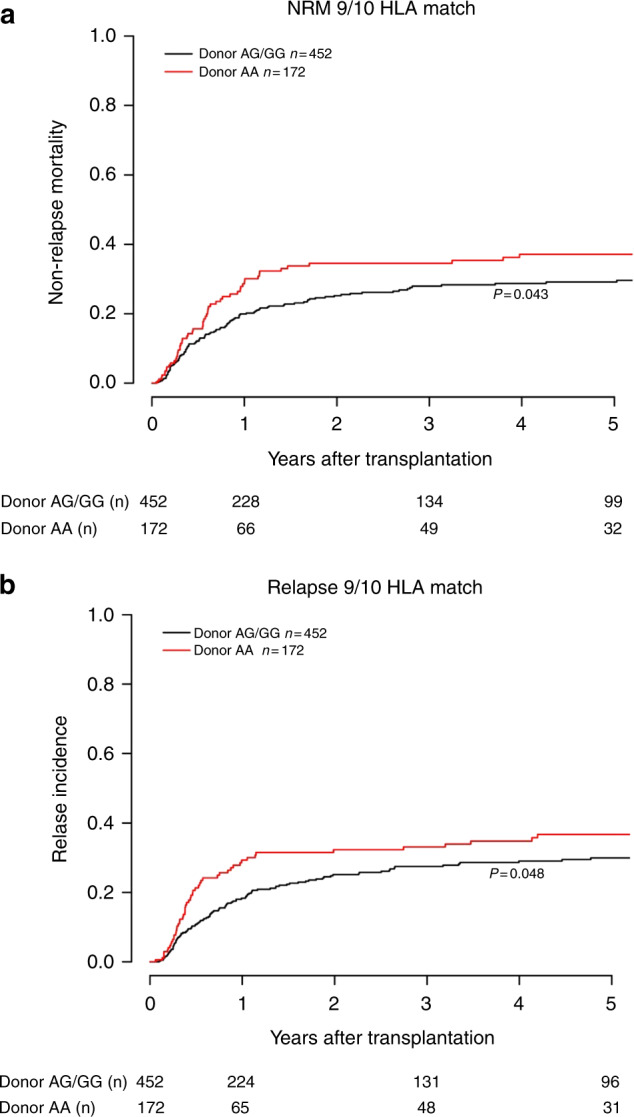
Univariate competing risks endpoints. **a** Non-relapse mortality according to donor rs2204985 in the subgroup of 9/10 HLA matched transplant pairs (*p* = 0.043). **b** Relapse incidence according to donor rs2204985 in the subgroup of 9/10 HLA matched transplant pairs (*p* = 0.048).

Analysis of the secondary clinical endpoints NRM and RI revealed that the adverse effect of donor AA genotype on survival was driven by a combined higher risk of RI (1Y after HSCT: 29.3% vs 18.3%; 5Y after HSCT: 36.7% vs 29.9%, *p* = 0.048) and NRM (1Y after HSCT: 28.6% vs 19.9%; 5Y after HSCT: 37.1% vs 29.1%, *p* = 0.043). Similar results were seen in the multivariate analyses for the two respective endpoints. No association was found between donor rs2204985 genotype and risk of acute or chronic GvHD. The results of the univariate analyses for NRM and RI are displayed in Fig. 3a, b, respectively. In Table 3 are summarized the results of the NRM, RI, aGvHD and cGvHD multivariate models.

**Table 3:** Competing risks endpoints 9/10 HLA-match.

	NRM		aGvHD		cGvHD		Relapse	
	HR (95% CI)	*P*-value	HR (95% CI)	*P*-value	HR (95% CI)	*P*-value	HR (95% CI)	*P*-value
Patient age	1.03 (1.02–1.04)	**<0.001**					1.03 (1.01–1.04)	**<0.001**
Donor age 18–30					1.00			
Donor age 31–45					1.41 (1.04–1.92)	**0.027**		
Donor age 46–60					0.99 (0.65–1.5)	0.954		
Early stage disease			1.00				1.00	
Intermediate stage disease			0.83 (0.61–1.12)	0.228			1.32 (0.92–1.88)	0.132
Advanced stage disease			1.34 (0.97–1.86)	0.081			1.60 (1.09–2.35)	**0.016**
rs2204985 donor AG/GG	1.00		1.00		1.00		1.00	
rs2204985 donor AA	1.38 (1.01–1.88)	**0.042**	0.88 (0.66–1.17)	0.371	1.12 (0.85–1.47)	0.425	until d300 1.74 (1.16–2.22), after d300 1.29 (0.74–2.22)	**0.007**, 0.368
HLA-DPB1 match/permissive MM	1.00		1.00					
HLA-DPB1 non-permissive MM	1.50 (1.13–2.00)	**0.005**	1.47 (1.14–1.89)	**0.003**				
In-vivo T-cell depletion			1.00					
No in-vivo T-cell depletion			1.44 (1.06–1.95)	**0.018**				
Year of transplantation 2000–2003	1.00							
Year of transplantation 2004–2009	0.32 (0.15–0.68)	**0.003**						
Year of transplantation 2010–2013	0.36 (0.16–0.8)	**0.012**						
RIC					1.00		1.00	
MAC					0.63 (0.48–0.81)	**<0.001**	1.58 (1.08–2.30)	**0.018**
KPS < 80	1.00						1.00	
KPS 80–100	0.53 (0.30–0.94)	**0.030**					0.56 (0.28–1.11)	0.096

Statistical significance is marked in bold.

*NRM* non-relapse survival, *aGvHD* acute graft versus host disease, *cGvHD* chronic graft versus host disease, *M* match, *MM* mismatch, *RIC* reduced intenstiy conditioning, *MAC* myeloablative conditioning, *KPS* Karnofsky performance score.

## Discussion

Optimal T cell immune reconstitution after HSCT is decisive for clinical success, as impaired thymic recovery has long been associated with increased risk of opportunistic infections, transplant-related morbidity and recurrence of primary disease [7]. It was only a few years ago that thymus function was found to be genetically predetermined by a common SNP, namely the rs2204985, located in the intergenic region of the TCRA-TCRD locus [11]. Intuitively one would wonder if this genetic factor could also play a role in an HSCT setting. The findings of the same research group appear to support this notion as the graft rs2204985 genotype was found to significantly correlate with post-transplantation thymic output in a mouse/human-HSC transplantation model [11]. Specifically it was shown that the graft rs2204985 AA genotype associated with inferior thymic output compared to the other two (i.e. AG and GG). In this study we sought to investigate this parameter in a human HSCT setting through a retrospective analysis of 2016 patients, who received their first unrelated allogeneic transplant between 2000 and 2013.

While analysis of the combined cohort did not confirm the initial hypothesis, subanalysis on account of HLA compatibility revealed that donor rs2204985 genotype was a significant predictive factor of outcome in single HLA mismatched cases. In contrast, no significant impact was identified on any outcome endpoint in the 10/10 HLA matched subgroup. One hypothesis for this difference observed between these two cohort subgroups could be that this effect becomes more relevant in an already endangered thymic milieu. It has been repeatedly reported that HLA mismatched HSCTs associate with a higher risk of GvHD and transplant-related mortality [21, 22]. This in turn is known to impair the thymic output either through direct attack on the thymic epithelium or indirectly through a secondary T cell immunodeficiency caused by a more intensive immunosuppression regimen [23, 24]. This compromised T cell recovery is believed to be at least partially responsible for the higher RI and NRM rates observed in HLA mismatched compared to HLA matched uHSCTs [6, 7]. Under this prism, it is plausible to postulate that the impact of this thymopoiesis-associating genetic marker may be more pronounced in an HSCT setting, where thymic function is at higher risk as already mentioned above. Our subanalysis on account of patient’s age supports this notion, as the donor’s rs2204985 genotype effect was only detectable—and in fact even more pronounced as compared to the whole cohort—in the older (i.e. ≥35 y) subgroup of patients. Although the number of cases in the younger cohort precludes a strong statistical power for this analysis, it does not cease to be an interesting finding that merits further investigation in the future. Furthermore, it is of note that direct comparison of survival with regard to donor rs2204985 genotype and HLA mismatch (Fig. 3 of Supplementary Data) revealed that donor AG/GG genotype abrogated to a great extent the detrimental impact of the single HLA mismatch. It should be also noted, that another subanalysis with respect to patient’s gender revealed that the donor rs2204985 genotype effect was more prominent in the male subgroup. This finding supports furthermore our hypothesis, that the donor rs2204985 genotype is mainly relevant in a compromised thymus function milieu (i.e. older age, male sex and single HLA mismatched HSCT).

Another important finding of this study was that the detrimental effect of donor AA genotype appeared to be conferred by a combined higher risk of NRM and RI. Given that no differences regarding the incidence of GvHD were observed with respect to this parameter, it is reasonable to assume that higher infection rates may account for the increased NRM. Although, we have no complete data regarding infection incidence, the higher NRM and relapse rates observed in patients receiving AA grafts are consistent with the assumption that this genotype adversely impacts the T cell recovery. Last, GvHD and especially its chronic form have been found to correlate with severe T cell immunodeficiency [24–26]. In our analysis the AA donor genotype did not correlate with significantly higher risk of cGvHD. However, given the relatively high percentage of missing data regarding this analysis, this aspect needs to be further clarified in future independent studies. On the other hand, the fact that better relapse control in patients transplanted with AG/GG grafts did not translate into higher aGvHD incidence rates is suggestive of an overall better T cell reconstitution with a broader but also more self-tolerant repertoire [6, 7]. Although human and mouse models are not one-to-one comparable, this hypothesis is supported by the findings of Clave et al. [11] already mentioned above. The exact mechanism through which the donor rs2204985 exerts its effect remains unclear. It seems, however, that the G allele correlates with superior thymic function, which in turn allows for a more complete and efficient immune reconstitution. This hypothesis is further supported by another mouse HSCT model, where it was shown that better preserved thymic function after HSCT correlated with superior immune reconstitution and decreased incidence of early post-transplantation adverse events [27]. It would be certainly interesting to investigate in future studies how this donor genetic marker may impact outcome after haploidentical HSCT as well as to what extent it might be relevant for pediatric patients, as both these HSCT settings exhibit distinct immunological features from the HSCT setting reviewed in this study.

Limitations of this study constitute missing data regarding infection incidence rates as well as actual measurement of T cell reconstitution surrogate markers like sjTRECs or βTRECs in patients before and after HSCT. Missing data was present for CMV status (see Table 1), blood group (2.2%) as well as the date of development of acute and chronic GvHD (aGvHD: 0.6%, cGvHD: 25.9%), limiting particularly the analysis for the endpoint cGvHD incidence. Another limitation is that patients not achieving complete remission were excluded, so our results are only representative for this subgroup of patients. Furthermore, our cohort represents patients transplanted in Germany and shows a large proportion of patients treated with ATG as part of the conditioning treatment as well as a low proportion of patients treated with tacrolimus based immunosuppression, which may limit future comparability with other cohorts showing different features. Last, another limitation of the study is that the limited cohort size of the 9/10 HLA matched subgroup precluded a comprehensive subanalysis statistical analysis on whether distinct HLA-locus mismatches differentially influenced the donor rs2204985 genotype effect. In our dataset, no statistically significant interactions were identified for these variables in the respective multivariate model (data not shown).

In conclusion this is to our knowledge the first study to date investigating the potential role of the donor genetic determinant of thymopoiesis, rs2204985, in the outcome of patients receiving unrelated HSC grafts. Our data suggest that donor rs2204985 AA genotype in combination with single HLA mismatches may adversely affect the outcome of HSC transplanted patients and should therefore be avoided. Older male patients receiving single HLA-mismatched HSC grafts are expected to benefit the most from such optimized donor selection. It is of note that one in four unrelated donors of Caucasian origin is expected to carry the AA genotype. A weaker relapse and –presumably- infection control due to compromised T cell reconstitution as a result of the unfavorable donor AA genotype may account for these findings. Confirmatory studies in larger independent cohorts are warranted before final conclusions are drawn.

## Supplementary information

Supplement

## Data Availability

The data that support the findings of this study are available from the corresponding author upon reasonable request after approval by the German Registry for Stem cell Transplantations (DRST).
