# Supplementary material for: Donor genetic determinant of thymopoiesis rs2204985 impacts clinical outcome after single HLA mismatched hematopoietic stem cell transplantation
Source: Bone Marrow Transplant. 2022 Jul 8;57(10):1539–47. doi: 10.1038/s41409-022-01751-1 (PMC9532242; doi:10.1038/s41409-022-01751-1)
Supplement: Supplementary file 1 — Supplement [file 41409_2022_1751_MOESM1_ESM.docx]

**Supplemental Material**


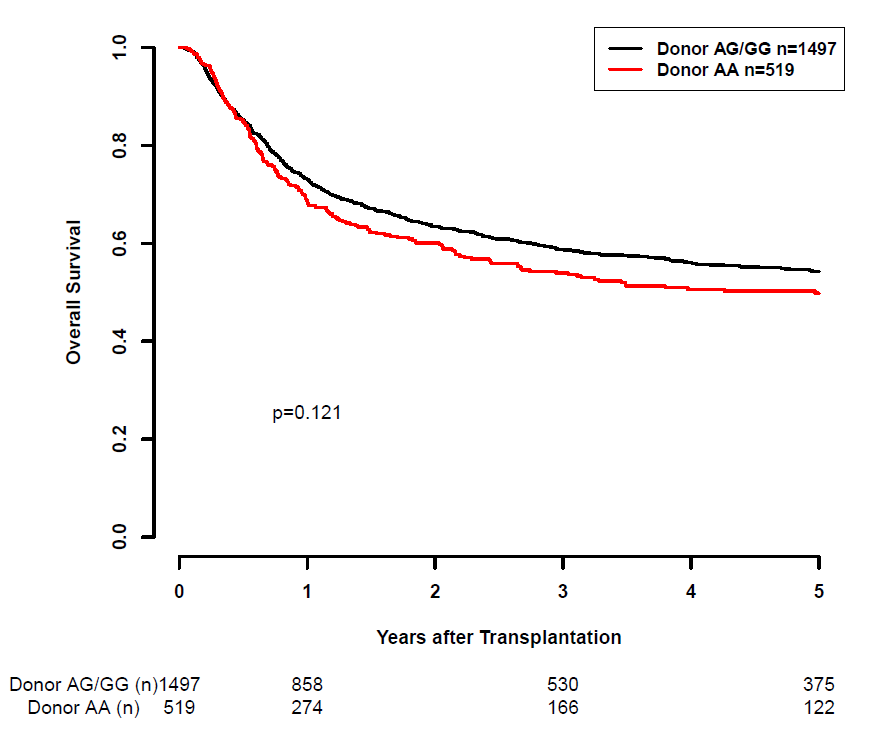


Supp. 1: OS with respect to donor rs2204985 in the combined cohort


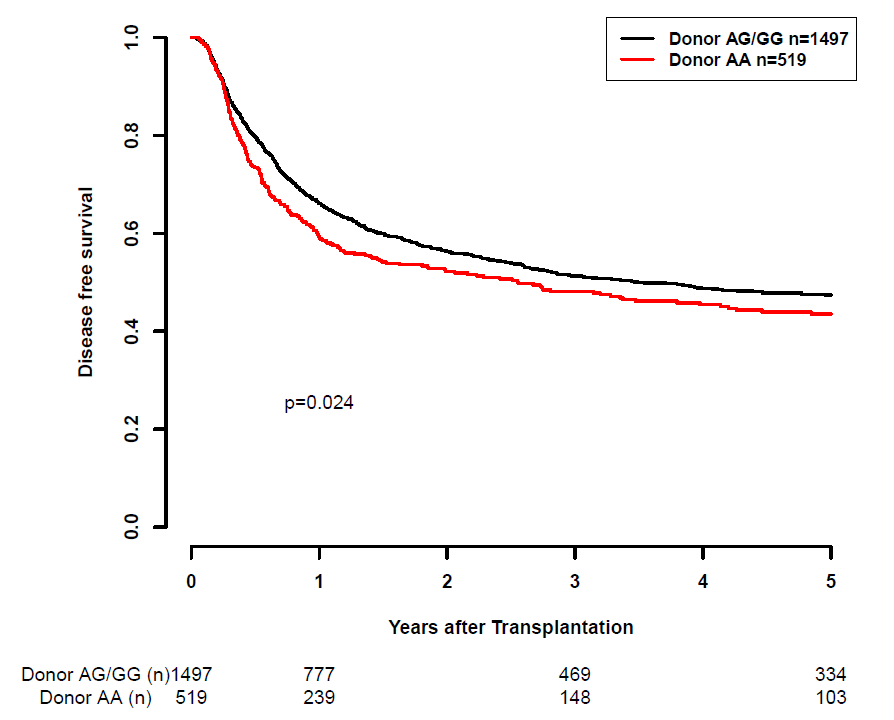


Supp. 2: DFS with respect to donor rs2204985 genotype in the combined cohort


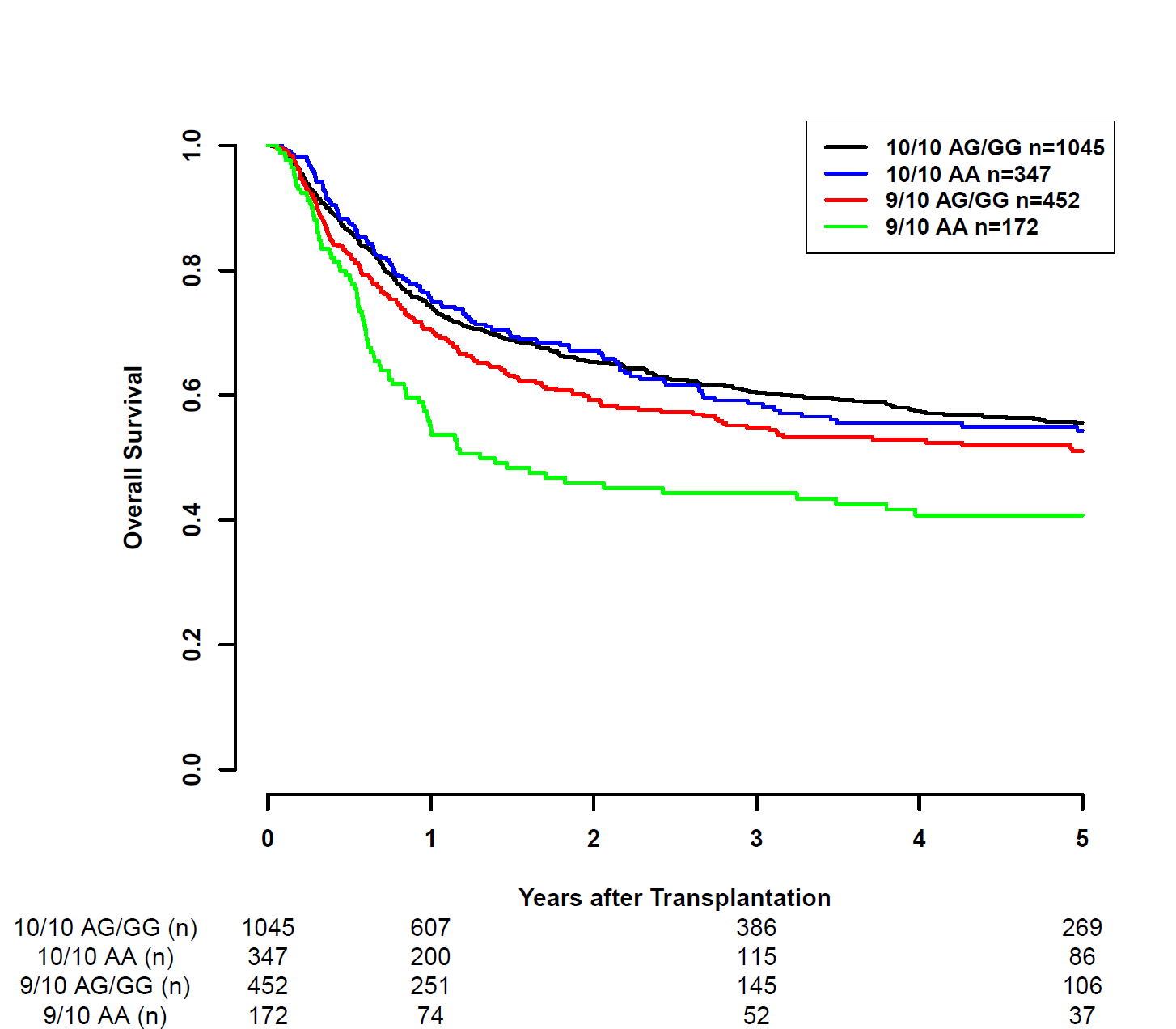


Supp. 3: Survival analysis with respect to donor rs2204985 genotype and HLA compatibility


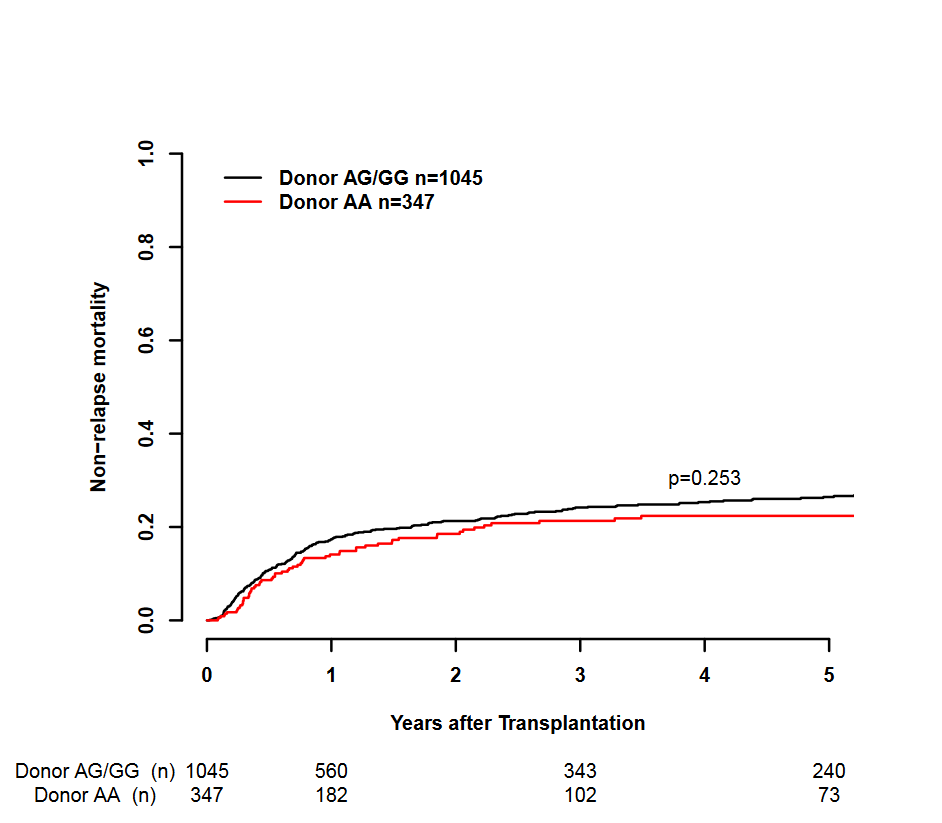


Supp. 4: NRM with respect to donor rs2204985 genotype in the 10/10 HLA matched subgroup


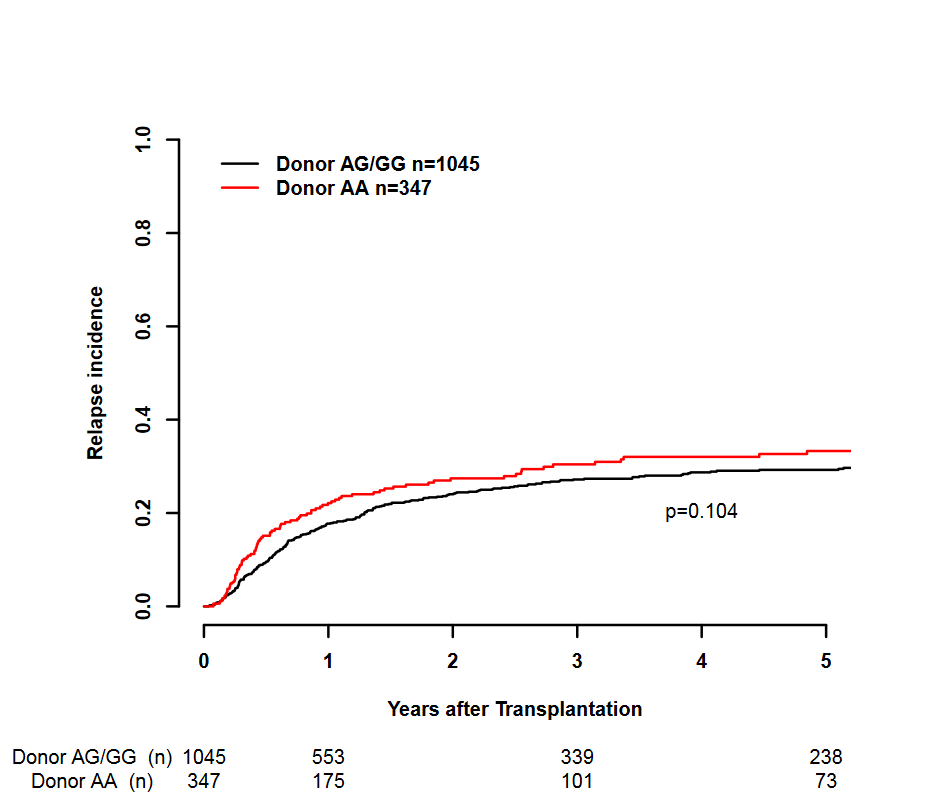


Supp. 5: Relapse incidence with respect to donor rs2204985 genotype in the 10/10 HLA matched subgroup


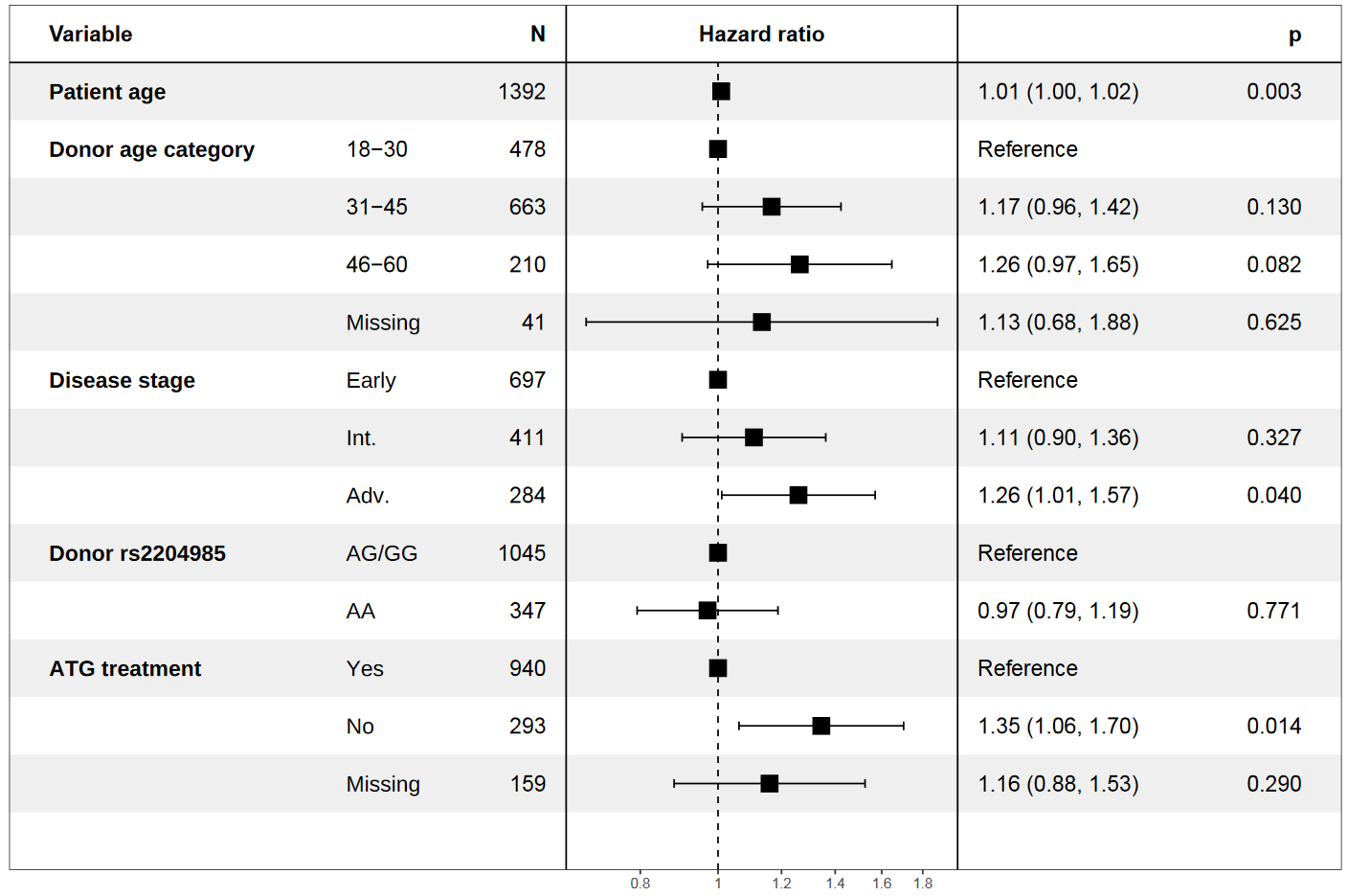


Supp. 6: Forest plot of multivariate analysis for overall survival in the 10/10 HLA matched subgroup


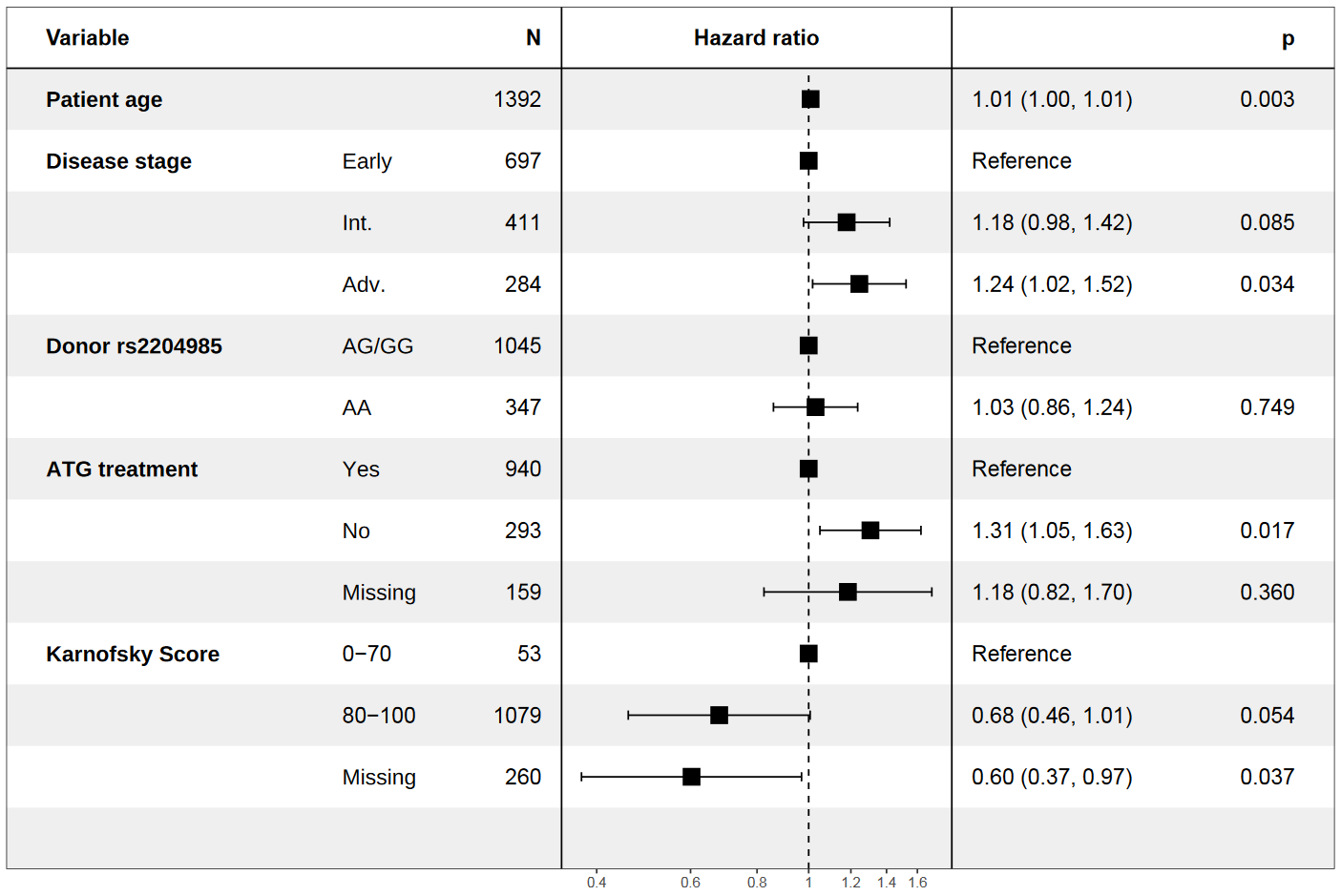


Supp. 7: Forest plot of multivariate analysis for disease free survival in the 10/10 HLA matched subgroup


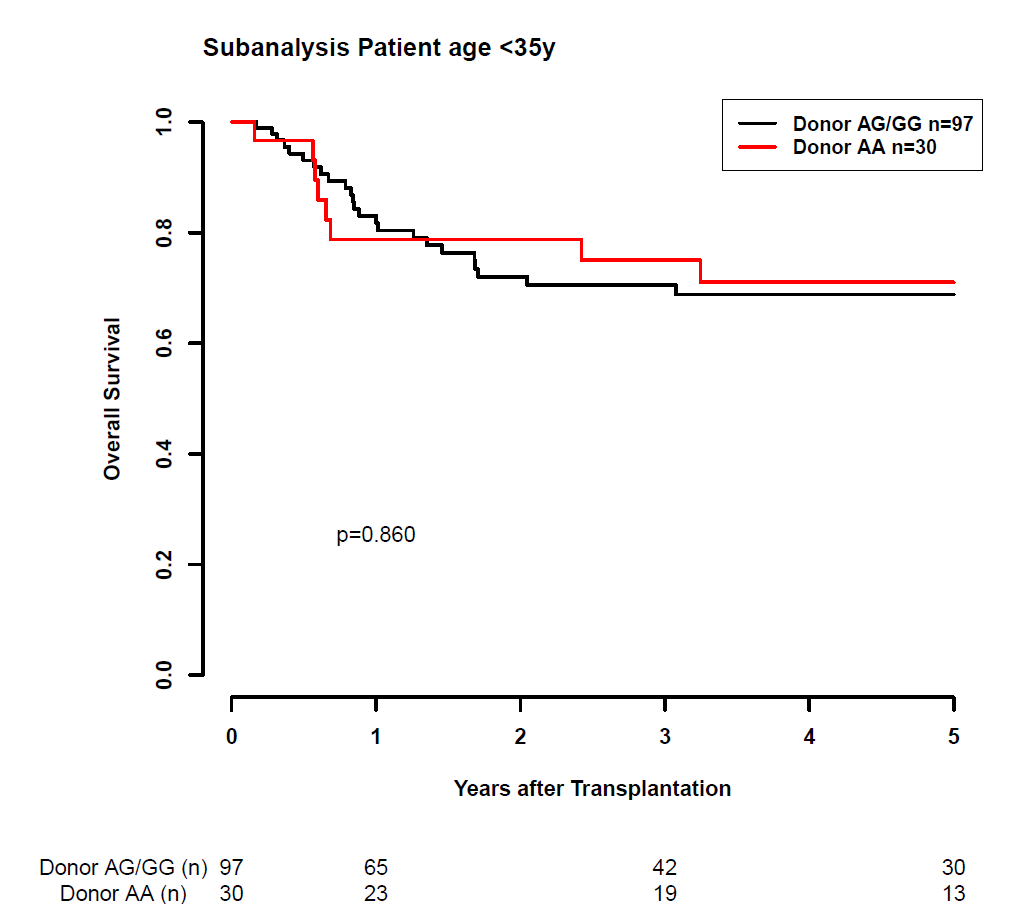


Supp. 8: OS with respect to donor rs2204985 genotype in the 9/10 HLA matched and <35y subgroup of patients


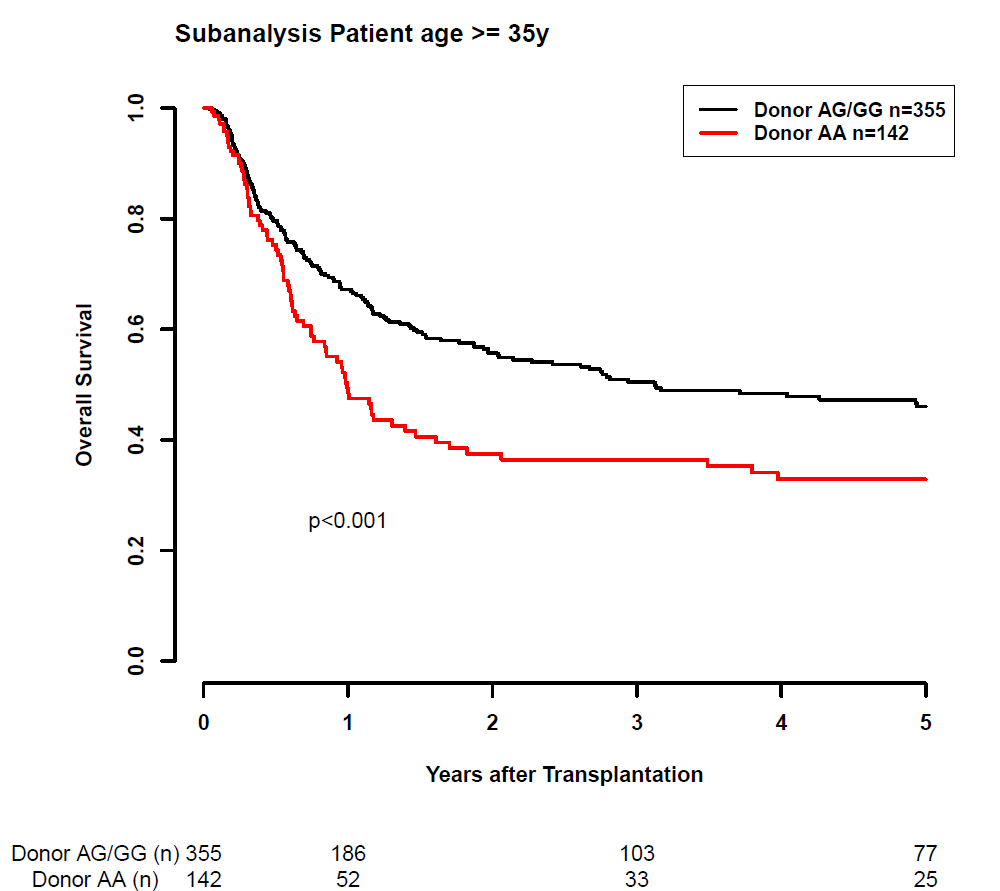


**≥**

Supp. 9: OS with respect to donor rs2204985 genotype in the 9/10 HLA matched and ≥35y subgroup of patients

| **Supplement table 1: Competing risks endpoints for 10/10 matched transplantations** | | | | | | |  |  |
| --- | --- | --- | --- | --- | --- | --- | --- | --- |
|  | **NRM** | | **aGvHD** | | **cGvHD** | | **Relapse** | |
|  | HR (95% CI) | p-Value | HR (95% CI) | p-Value | HR (95% CI) | p-Value | HR (95% CI) | p-Value |
| **Patient age** | 1.01 (1.01-1.02 | **<0.001** | - |  | - |  | - |  |
| **Donor age 18-30** | 1.00 |  | 1.00 |  | 1.00 |  | - |  |
| **Donor age 31-45** | 1.19 (0.92-1.53) | 0.182 | 1.26 (1.00-1.60) | 0.055 | 1.09 (0.88-1.35) | 0.409 | - |  |
| **Donor age 46-60** | 1.37 (0.99-1.9) | 0.061 | 1.43 (1.06-1.94) | **0.020** | 1.28 (0.97-1.69) | 0.077 | - |  |
| **Early stage disease** | - |  | 1.00 |  | - |  | - |  |
| **Intermediate stage disease** | - |  | 1.26 (1.00-1.58) | **0.050** | - |  | - |  |
| **Advanced stage disease** | - |  | 1.09 (0.83-1.42) | 0.541 | - |  | - |  |
| **rs2204985 Donor AG/GG** | 1.00 |  | 1.00 |  | 1.00 |  | 1.00 |  |
| **rs2204985 Donor AA** | 0.87 (0.67-1.13) | 0.313 | 0.86 (0.68-1.10) | 0.227 | 0.82 (0.66-1.03) | 0.086 | 1.19 (0.95-1.49) | 0.125 |
| **HLA-DPB1 Match/Permissive** | - |  | 1.00 |  | 1.00 |  | - |  |
| **HLA-DRB1 Non-Permissive** | - |  | 1.36 (1.11-1.67) | **0.003** | 1.17 (0.97-1.41) | 0.095 | - |  |
| **In-vivo T-cell depletion** | 1.00 |  | 1.00 |  | 1.00 |  | - |  |
| **No in-vivo T-cell depletion** | 1.66 (1.26-2.18) | **<0.001** | 1.59 (1.27-2.01) | **<0.001** | 1.82 (1.49-2.23) | **<0.001** | - |  |
| **RIC** | 1.00 |  | - |  | - |  | - |  |
| **MAC** | 1.35 (1.04-1.74) | **0.024** | - |  | - |  | - |  |
| **KPS <80** | - |  | - |  | - |  | 1.00 |  |
| **KPS 80-100** | - |  | - |  | - |  | 0.61 (0.37-1.00) | **0.050** |
| Abbrevations: NRM=Non-relapse survival, aGvHD=Acute Graft versus Host disease, cGvHD=Chronic Graft versus Host disease, M=Match, MM=Mismach, RIC=Reduced intenstiy conditioning, MAC=Myeloablative conditioning, KPS=Karnofsky Performance Score. Statistical significance is marked in bold | | | | | | | | |

| **Supplement table 2A: OS Subanalysis Male Patients Only** | | |
| --- | --- | --- |
|  | **OS** | |
|  | HR (95% CI) | P-value |
| **Patient age** | 1.03 (1.02-1.05) | **<0.001** |
| **rs2204985 Donor AG/GG** | 1.00 |  |
| **rs2204985 Donor AA** | 1.76 (1.27-2.43) | **0.001** |
| **RIC** | 1 |  |
| **MAC** | 1.55 (1.09-2.22) | **0.015** |
| **KPS <80** | 1 |  |
| **KPS 80-100** | 0.29 (0.15-0.55) | **<0.001** |
| Abbrevations: OS=Overall survival, RIC=Reduced intenstiy conditioning, MAC=Myeloablative conditioning, KPS=Karnofsky Performance Score. Statistical significance is marked in bold | | |

| **Supplement table 2B: OS Subanalysis Female Patients Only** | | |
| --- | --- | --- |
|  | **OS** | |
|  | HR (95% CI) | P-value |
| **Patient age** | 1.03 (1.02-1.05) | **<0.001** |
| **rs2204985 Donor AG/GG** | 1.00 |  |
| **rs2204985 Donor AA** | 1.22 (0.8-1.85) | 0.364 |
| **RIC** | 1 |  |
| **MAC** | 1.55 (0.98-2.44) | 0.061 |
| **KPS <80** | 1 |  |
| **KPS 80-100** | 0.46 (0.23-0.92) | **0.027** |
| Abbrevations: OS=Overall survival, RIC=Reduced intenstiy conditioning, MAC=Myeloablative conditioning, KPS=Karnofsky Performance Score. Statistical significance is marked in bold | | |
